# Single Level Spondylolisthesis Associated Sagittal Plane Imbalance Corrected by Pre-Psoas Interbody Fusion Using Anterior Column Release with 30° Expandable Hyperlordotic Cage

**DOI:** 10.3390/medicina58091172

**Published:** 2022-08-29

**Authors:** Mansour Mathkour, Stephen Z. Shapiro, Tyler Scullen, Cassidy Werner, Mitchell D. Kilgore, Velina S. Chavarro, Daniel R. Denis

**Affiliations:** 1 Department of Neurological Surgery, Ochsner Health, New Orleans, LA 70121, USA; 2 Department of Neurosurgery, Northwell Health, Manhasset, NY 11030, USA; 3 Faculty of Medicine, University of Queensland, Brisbane, QLD 4029, Australia

**Keywords:** sagittal plane imbalance, single-level spondylolisthesis, minimally invasive anterior column release, expandable hyperlordotic cage, anterior to psoas interbody fusion

## Abstract

*Background:* Loss of lumbar lordosis caused by single level degenerative spondylolisthesis can trigger significant sagittal plane imbalance and failure to correct lumbopelvic parameters during lumbar fusion can lead to poor outcome or worsening deformity. Anterior column release (ACR) through a pre-psoas approach allows the placement of a hyperlordotic cage (HLC) to improve lumbar lordosis, but it is unclear if the amount of cage lordosis affects radiological outcomes in real-life patient conditions. *Methods:* Three patients were treated with ACR and 30° expandable HLC for positive sagittal imbalance secondary to single-level spondylolisthesis. Patients reported baseline and post-operative Oswestry Disability Index (ODI) and Numeric Pain Score (NRS). Radiographic parameters of sagittal balance included lumbar lordosis (LL), sagittal vertical axis (SVA) and pelvic incidence-lumbar lordosis mismatch (PI-LL). *Results:* Surgical indications were sagittal plane imbalance caused by L4–L5 degenerative spondylolisthesis (n = 2) and L3–L4 spondylolisthesis secondary to adjacent segmental degeneration (n = 1). Average post-operative length of stay was 3 days (range 2–4) and estimated blood loss was 266 mL (range 200–300). NRS and ODI improved in all patients. All experienced improvements in LL ($\bar{x}$preop = 33°, $\bar{x}$postop = 56°), SVA ($\bar{x}$preop = 180 mm, $\bar{x}$postop = 61 mm) and PI-LL ($\bar{x}$preop = 26°, $\bar{x}$postop = 5°). *Conclusion:* ACR with expandable HLC can restore sagittal plane balance associated with single-level spondylolisthesis. Failure to perform ACR with HLC placement during pre-psoas interbody fusion may result in under correction of lordosis and poorer outcome for these patients.

## 1. Introduction

Degenerative spondylolisthesis in the lumbar spine can be associated with dynamic instability and spinal stenosis causing refractory low back pain, leg pain and decreased functional status [1,2]. Sagittal plane imbalance associated with single level degenerative spondylolisthesis is not a well-studied phenomenon, and tends to be overlooked, unless scoliosis films are systematically analyzed in this patient group [3]. Complications arising from lumbar fusion for spondylolisthesis, such as flat back surgery syndrome, adjacent segment degeneration and failed back surgery syndrome, may arise from under correction of sagittal plane imbalance and the failure of increasing global lumbar lordosis (LL) [4,5,6]. Traditionally, restoration of sagittal plane balance requires extensive posterior long-segment spinal instrumented fusion (PSIF), with nearly 44% of patients receiving fusion of over four levels [7]. Unfortunately, long segment PSIF is associated with proximal junctional kyphosis (PJK) or adjacent segment degeneration from secondary stress centered at both ends of the instrumentation where torque is greatest and is particularly prevalent in patients with sagittal imbalance [8].

Anterolateral or direct lateral approaches to the lumbar spine involving anterior column release (ACR) and interbody fusion provide a relatively noninvasive route to correct LL and restore sagittal balance while mitigating the morbidity of the posterior approach [9]. ACR may be accomplished via a minimally invasive (MIS) direct lateral or transpsoas route to the lumbar spine above the lumbosacral junction [10]. This modality has demonstrated a 10–27° increase in segmental lordosis, 16° to 31° increase in global lumbar lordosis, with a 5.3% risk of PJK. Possible complications from the transpsoas route include lumbosacral plexus injury causing post-operative thigh pain or dysesthesia [11]. Motor complications are also commonly encountered, such as hip flexion weakness from direct psoas muscle injury or prolonged femoral nerve retraction [12]. Alternatively, the anterolateral or pre-psoas approach provides a natural corridor to the left anterolateral aspect of the lumbar disc spaces from L2–S1 without division of the major psoas muscles, and a significant decrease in motor and sensory complications [13]. The corridor is particularly useful for accessing the L4–L5 interspace, for which access is often hindered by iliac crest obstruction and the progressively anterior location of the lumbosacral plexus. The pre-psoas approach allows placement of large hyperlordotic cages (HLC), which can provide up to 29° of segmental lordosis [14], allowing an alternative to the historic complex open corrections for deformity. A series of three patients, with single level spondylolisthesis associated with sagittal plane imbalance, who underwent correction of sagittal plane imbalance using a 30° HLC through a pre-psoas approach is presented.

## 2. Report of Cases

For this case series, an electronic medical record review of all patients with positive sagittal imbalance caused by single level spondylolisthesis was carried out. Patients treated with ACR and pre-psoas interbody fusion, using the fully expanded 30° interbody spacer ELSA^®^, Globus Medical (Audubon, PA, USA), by a single surgeon (DD) at our academic tertiary care center from April 2019 through August of 2022 were included. For comparison, two cases having sagittal plane imbalance and single level spondylolisthesis treated with pre-psoas interbody fusion, without ACR and with a lesser lordotic cage, were included. Sagittal plane imbalance was assessed on lateral scoliosis radiographs using the C7 sagittal vertical axis (SVA) technique [9]. Positive sagittal plane imbalance was defined as SVA greater than 7 cm. Demographic and clinical data, including body mass index (BMI), operative time (ORT), estimated blood loss (EBL), and hospital length of stay (LOS) are reported. Patient functional outcomes, including Oswestry Disability Index (ODI) and Numeric Pain Score (NRS), were measured at baseline and at 2, 6, 12, 24, and 36 weeks follow-up. Baseline and post-operative radiographic parameters, SVA, global lumbar lordosis (LL) and pelvic incidence-lumbar lordosis mismatch (PI-LL) were collected utilizing SurgimapTM (New York, NY, USA). Study outcomes were examined using observational statistics.

### 2.1. Surgical Approach

Careful pre-operative review of lumbar spine imaging, including computed tomography (CT), magnetic resonance imaging (MRI), and scoliosis radiographs, help localize the large vessels and evaluate relevant parameters of spinal deformity. After general anesthesia and intubation, patients are placed in a lateral decubitus position with the left side up on a sliding table. All pressure points are carefully padded. Fluoroscopic is utilized to identify the intended level. Electromyogram (EMG) and somatosensory evoked potentials (SSAP) are monitored. An oblique incision measuring 3–5 cm is made according to the intended level. The subcutaneous tissue is dissected bluntly, and a corridor is extended through the external oblique, internal oblique, and transversus abdominis musculature aiming dorsally and towards the anterior superior iliac crest. The retroperitoneal space is dissected bluntly anterior to the psoas and the spine. A radiopaque probe is placed on a trajectory for the intended level. Serial dilators are then inserted over the probe and a self-sustaining retractor system is secured in place just anterior to the psoas major muscle, centered on the intended disc space. Position is confirmed with biplanar fluoroscopy. The middle blade is oriented anteriorly, leaving sufficient space between the caudal and cranial blades for instruments to be freely manipulated.

Annulotomy is performed using angled Cobb elevators and the contralateral annulus is divided under fluoroscopic guidance. Discectomy is completed using serial shavers and pituitary rongeurs. The endplates are carefully scraped of disc material using a down pushing curette. A trial cage is inserted, and its length is measured on anteroposterior view fluoroscopy. An expandable 30° HLC, packed with viable cell allograft, TrifectaTM (Globus Medical, Audubon, PA, USA) is then inserted under fluoroscopy and partially expanded. Integral instrumentation is performed with one screw in the vertebral body above and below the disc space to stabilize the spacer. A manual blade retractor is bluntly placed anterior to the disc space to protect the iliac vessels, ureter, and peritoneum. ACR is accomplished via sharp division of the anterior longitudinal ligament (ALL) under direct visualization. The cage is then fully expanded to 30° and post-filled with more allograft. Following hemostasis, the retroperitoneal space is closed by suturing the external oblique fascial plane. The patient is then transferred prone on a Jackson table and posterior instrumentation is carried out using pedicle screws and rods placement.

### 2.2. ACR Cases Demographic and Pre-Operative Outcome

Three cases who underwent ACR and pre-psoas interbody fusion with a 30° expandable HLC are included in this report. Two cases who underwent pre-psoas interbody fusion without ACR are presented for comparison. Age at surgery for the ACR treated cases was 63 years (range 58–67) and average BMI was 40.3 kg/m^2^ (range 27–49) (Table 1). Indication for the procedure was L4–L5 Grades I and II degenerative spondylolisthesis in two patients, and L3–L4 Grade I spondylolisthesis secondary to adjacent segment disease in the third patient (Table 2). All patients had pre-operative sagittal plane imbalance (Table 3).

Average EBL was 267 mL (range 200–300 mL), ORT 185 min (range 170–195), and LOS 3 days (range 2–4) (Table 1). No associated complications (including cage subsidence) are reported.

### 2.3. ACR Cases Functional and Radiographic Outcome

**Figure 1:**
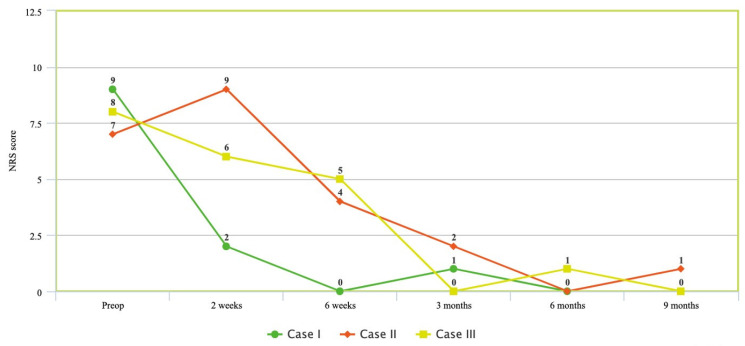
Numeric rating scale (NRS) changes over time for patients pre-operatively and at 2-week, 6-week, 3-month, 6-month, and 9-month follow-up.

**Figure 2:**
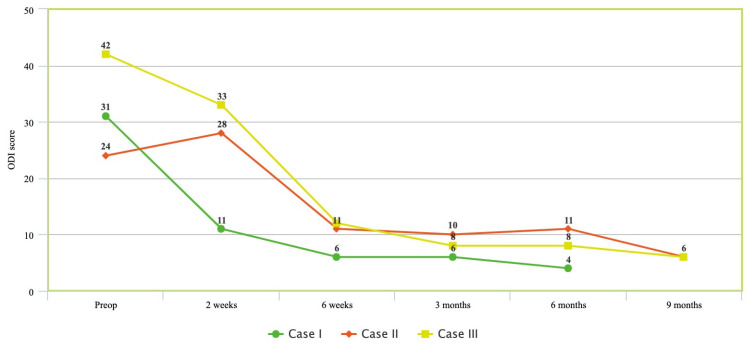
Oswetry disability index (ODI) changes over time for patients pre-operatively and at 2-week, 6-week, 3-month, 6-month, and 9-month follow-up.

Mean follow-up time was 8 months (range 6–9). No evidence of cage subsidence or lucency around hardware was noted at each follow-up visit on lumbar X-ray. All patients experienced improvements in NRS at 6-month follow-up; patients 2 and 3 additionally continued to display improvement at 9 months (Figure 1). Similarly, ODI measurements at follow-up improved in all patients across the same period (Figure 2). All patients experienced significant improvements in sagittal balance parameters including LL, SVA, and PI-LL (Table 3).

### 2.4. ACR Cases

#### 2.4.1. Case I

**Figure 3:**
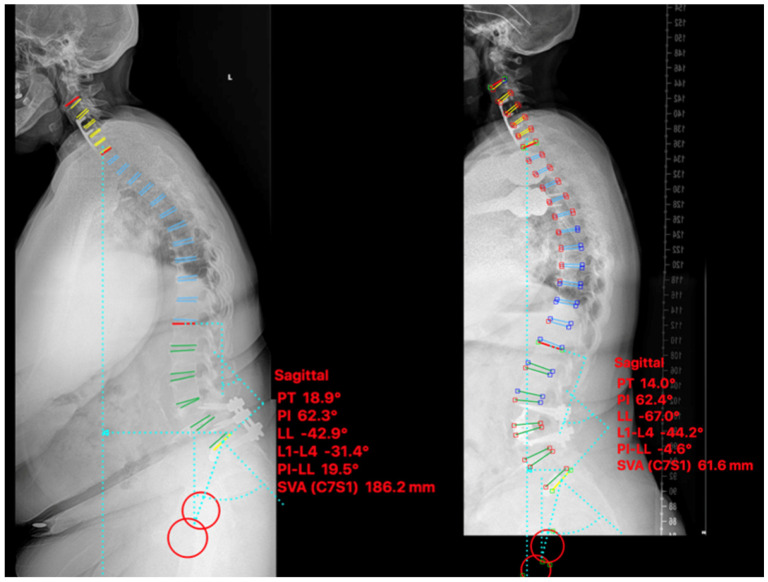
Case I. Pre-psoas interbody fusion for L3-4 spondylolisthesis with anterior column release and an expandable 30° cage. Lateral standing scoliosis radiographs. Pre-operative imaging (**left**), 6-months postoperative imaging (**right**).

A 67-year-old female with prior L4-S1 posterior instrumented fusion for spondylosis presented with clinical lumbar radiculopathy secondary to adjacent segment disease and L3-4 grade I anterolisthesis. There was significant spinal stenosis at L3-4 and sagittal plane imbalance was noted on imaging. The patient’s baseline NRS and ODI were 9 and 31 respectively, which improved to 0 and 4 at 6-month post-operatively. Pre-operative sagittal balance parameters were LL 43°, SVA 186 mm, and PI-LL 20° with improvement postoperatively to LL 67°, SVA 62 mm, and PI-LL −5° (Figure 3).

#### 2.4.2. Case II

**Figure 4:**
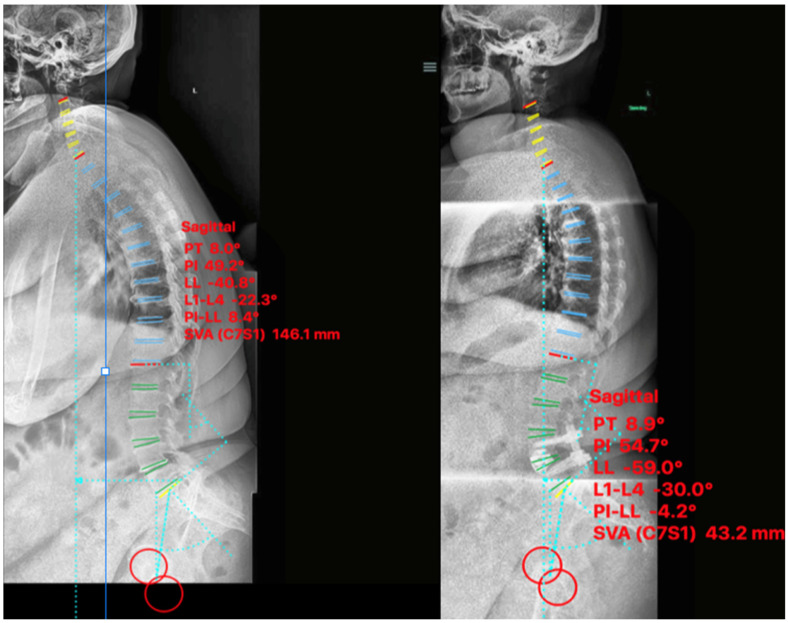
Case II. Pre-psoas interbody fusion for L4–5 spondylolisthesis with anterior column release and an expandable 30° cage. Lateral standing scoliosis radiographs. Pre-operative imaging (**left**), 3-months post-operative imaging (**right**).

A 64-year-old female with prior history of L4-L5 microdiscectomy for herniated nucleus pulposus presented with lumbar radiculopathy secondary to grade I L4-L5 degenerative spondylolisthesis with sagittal plane imbalance. The patient’s baseline NRS and ODI were 7 and 24 respectively, which improved to 1 and 6 at 9-month post-operatively. Pre-operative sagittal balance parameters were LL 41°, SVA 146 mm, PI-LL 8° with im-provement postoperatively to LL 59°, SVA 43 mm, PI-LL −4° (Figure 4).

#### 2.4.3. Case III

**Figure 5:**
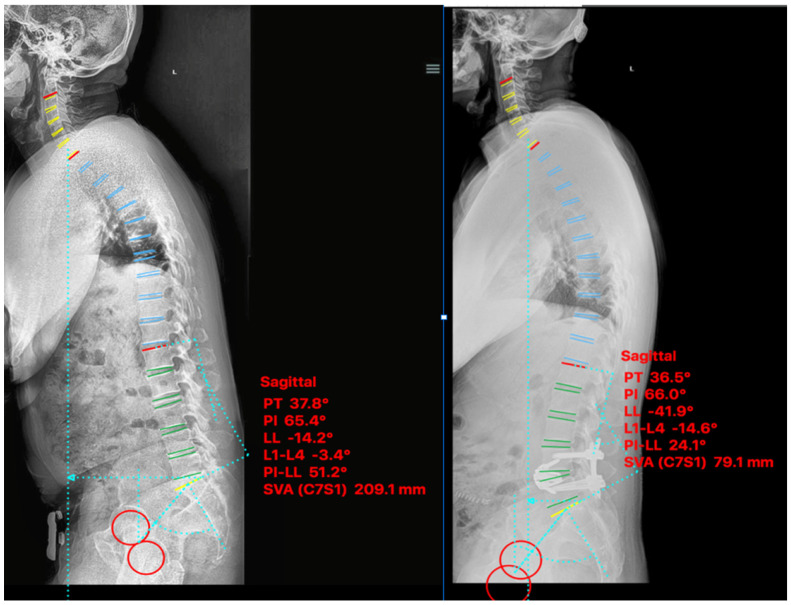
Case III. Pre-psoas interbody fusion for L4-5 spondylolisthesis with anterior column release and an expandable 30° cage. Lateral standing scoliosis radiographs. Pre-operative imaging (**left**), 1-month post-operative imaging (**right**).

A 58-year-old male presented with grade I L4-5 degenerative spondylolisthesis and sagittal plane imbalance. The patient’s baseline NRS and ODI were 8 and 42 respectively, which improved to 0 and 6 at 9-month follow up. Pre-operative sagittal balance parameters were LL 14°, SVA 209 mm, PI-LL 51° with improvement postoperatively to LL 42°, SVA 79 mm, PI-LL 24° (Figure 5).

### 2.5. Non-ACR Cases

#### 2.5.1. Case IV

**Figure 6:**
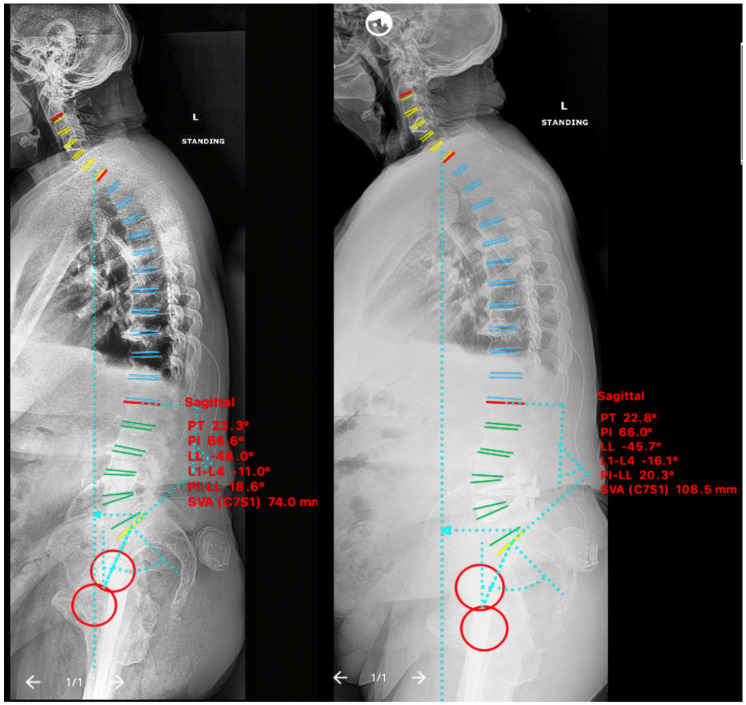
Case treated with pre-psoas interbody fusion for L4–5 spondylolisthesis without anterior column release and with an expandable 15° cage. Lateral standing scoliosis radiographs. Pre-operative imaging (**left**), 5 months post-operative imaging (**right**). Note the worsening of sagittal plane imbalance and pelvic-incidence mismatch parameters post-operatively.

An 85-year-old male presented with grade I L4-5 degenerative anterolisthesis and sagittal plane imbalance. A fully expanded growing lordosis 15° spacer was used, Rise-L®, Globus Medical (Audubon, PA, USA). The patient’s baseline NRS and ODI were 8 and 56 respectively. Post-operatively his severe leg pain improved but his back pain and ODI remained the same. Pre-operative sagittal balance parameters were LL 48°, SVA 74 mm, PI-LL 18° with worsening post-operatively to LL 46°, SVA 109 mm, PI-LL 20° (Figure 6).

#### 2.5.2. Case V

**Figure 7:**
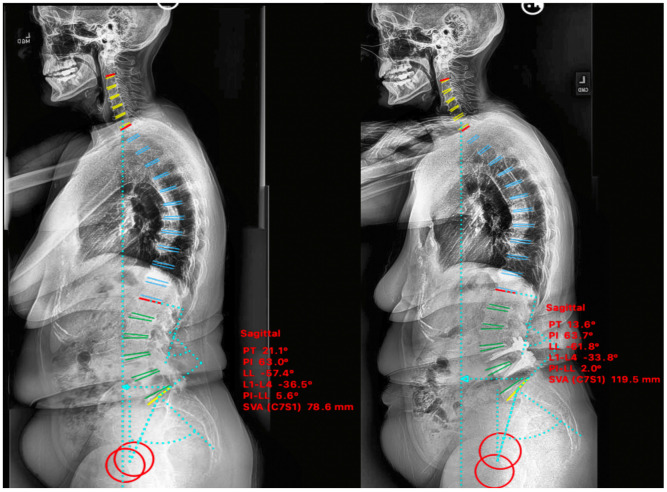
Case treated with pre-psoas interbody fusion for L4–5 spondylolisthesis without anterior column release and with a static titanium 8° cage. Lateral standing scoliosis radiographs. Pre-operative imaging (**left**), 3 months post-operative imaging (**right**). Note the worsening of sagittal plane imbalance but improvement of pelvic-incidence mismatch parameters post-operatively.

A 71-year-old female presented with grade II L4-5 degenerative anterolisthesis with severe spinal stenosis and sagittal plane imbalance. A static titanium spacer with 8° of lordosis was used, Conduit™, DePuy Synthes (Raynham, MA, USA). The patient’s baseline NRS and ODI were 8 and 29, respectively. Three months after surgery her NRS decreased to 2 and her ODI increased to 40. Pre-operative sagittal balance parameters were LL 57.4°, SVA 79 mm and PI-LL 6°. Post-operatively improvement of the LL 62° and PI-LL 2° but worsening of the SVA 120 mm was found (Figure 7).

## 3. Discussion

Biomechanical understanding of sagittal imbalance is paramount to understanding physiologic compensatory mechanisms and subsequent clinical manifestations. Ideal spinal alignment allows an individual to assume standing posture with minimal muscular energy expenditure [15]. Normally, this is accomplished through a complex relationship that exists between the physiologic curvatures of the spine, the morphology of the pelvis, and the musculature of the axial and appendicular skeleton. Increasing positive sagittal imbalance causes the body to assume a position toward the periphery of the Dubousset cone of economy, which results in increased muscular effort and energy expenditure causing pain, fatigue, and disability [16]. Glassman and colleagues [17] were the first to report on the detrimental pain and functional effects of sagittal imbalance greater than 4 cm and, similarly, Mac-Thiong et al. [18] attributed a positive imbalance greater than 6 cm to worse health–related quality of life scores as evidenced by ODI results.

### 3.1. Historical Treatment of Sagittal Plane Imbalance

Historically, sagittal plane imbalance has been managed with spinal osteotomies of varying extent depending on the intended degree of correction [9]. The amount of sagittal correction obtained from the less extensive Smith-Peterson Osteotomy (SPO) relies on flexibility of the disc space and can achieve approximately 10° of correction [9]. The more extensive pedicle subtraction osteotomy (PSO) can generate 20° to 40° of LL and an approximately 10–12 cm improvement in SVA depending on the wedge of bone removed [9]. Despite aggressive correction with PSO, sagittal decompensation assessed with SVA >8 cm may develop post-operatively in a subset of patients (29%) [9]. Additionally, these conventional procedures can be marked by high volumes of blood loss and surgical complications [19], as well as PJK [8].

Anterior column reconstruction using structural grafts, either through traditional anterior or anterolateral approaches or MIS direct lateral approaches, provide anterior column lengthening while precluding the wide exposure of posterior-based osteotomies [9]. Because 60% to 80% of anatomic LL is found between L4 to S1, improved outcomes are observed when the deformity correction and created LL are derived from these lower lumbar segments [20]. ACR accomplished via ALL release is inherent in these procedures and allows for maximal lordosis correction [20].

Typically, the lumbar spine has 60° of LL from T12-S1 [20], though there is an ideal correlation of LL with an individual’s PI when these parameters are lying within 9° of one another [16]. PI-LL mismatch and SVA are associated with adverse patient-reported outcomes [21,22]. Therefore, the goals of surgical correction involve optimizing PI-LL and SVA to achieve global sagittal balance [23].

### 3.2. Minimally Invasive Surgery

Circumferential MIS approaches are associated with decreased surgical complications [8,24,25]. Traditionally MIS techniques have been limited by insufficient correction of marked sagittal deformity [25]. The MISDEF2 algorithm, which was developed in October 2019, proposes MIS candidacy based on dynamicity of deformity, sagittal plane imbalance, and coronal deformity [26]. Specifically, patients with fused/rigid spines and LL-PI mismatch in excess of 30° can be effectively managed with circumferential MIS approaches, including ACR, expandable HLC, mini-open PSO.

With similar sagittal correction magnitude to the PSO, the anterior or lateral procedures involving ALL release and anterior column structural support are a predictable method to correct LL and restore sagittal balance while mitigating the morbidity of the posterior dissection [9]. Laterally based, MIS retroperitoneal transpsoas approaches for ACR can create 10.2° of segmental lordosis with operative times of less than one hour and EBL less than 50 mL [24]. MIS techniques involving a pre-psoas approach have been used with success in the placement of interbody fusions from T12-S1 [13,25]. The advantages of the pre-psoas approach include visualization of the retroperitoneal vasculature with avoidance of iatrogenic vessel, psoas and lumbar plexus injuries [25]. Pre-psoas approaches for ACR have also been described to provide effective routes of correction [27].

Successful restoration of sagittal plane imbalance using the transpsoas approach has been reported with no significant difference radiographically as compared to PSO and with significant decreases in EBL [11]. Barone et al. [28] reported a significantly decreased PI-LL of 8.2° to 4.2° and a non-significant increase in LL from 41.4° to 46.5° in a cohort of patients that underwent transpsoas HLC placement; however, they did not specify the number of those patients that additionally underwent ACR. Anterior insertion of 20° HLCs can achieve up to 19° of segmental lordosis, while 30° HLC can achieve up to 29° of segmental lordosis with an average blood loss of only 240 mL [14].

**Figure 8:**
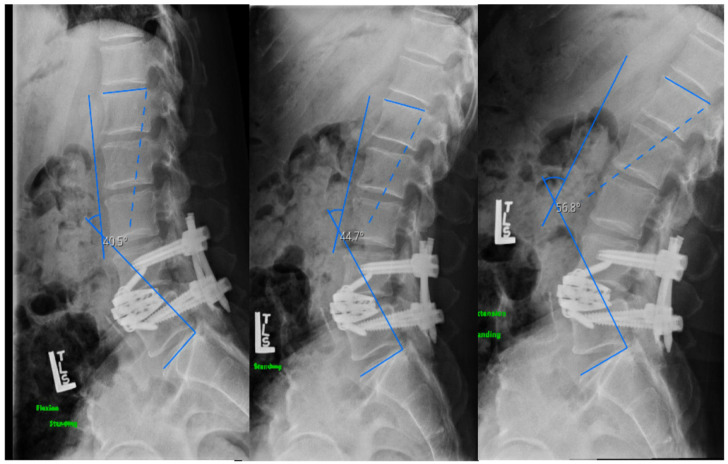
Effect of posture on lumbar lordosis in case III. Flexion-extension lumbar X-ray at 16-months post-operative showing more than 16 degrees of difference in lumbar lordosis between flexion and extension. Lumbar lordosis-pelvic incidence mismatch normalized with lumbar extension.

The effect of ACR combining cages with varying amounts of lordosis can be studied on biomechanical models [29], but there has been insufficient data on patients using 30° HLC for single-level spondylolisthesis. A mean increase of 23.3° in LL (range 18–28°) was found in the three cases of ACR with HLC presented in this study. Using a transpsoas approach, Barone et al. [28] achieved a mean increase in LL of 9°, while Xu et al. [10] were able to achieve an increase in mean LL of 16.7, though the latter study incorporated the use of posterior osteotomies in a subset of patients. The greater increase in average LL found in the present study can be explained by the use of fully expanded 30° HLC. Two of the ACR reported cases had PI-LL mismatch correction to less than 0°, which can be considered excessive but this was necessary in order to correct the significant sagittal plane imbalance in these patients. The remaining ACR case demonstrated correction of PI-LL mismatch from 51 to 24°. This is considered less than ideal but at 16 months postoperative, extension lumbar radiographs of this patient demonstrated a lumbar lordosis of 57° and a PI-LL mismatch of 9°. This demonstrates that LL and SVA varies substantially depending on posture of the flexible lumbar spine (Figure 8). Nevertheless, under correcting lumbar lordosis during lumbar fusion is thought to lead to suboptimal outcomes (Figure 6) and increased risk of developing adjacent segment degeneration [18,21,22]. The two non-ACR cases presented demonstrated suboptimal post-operative radiological results; their SVA increased post-operatively even if their pre-operative SVA was significantly inferior when compared to the ACR cases presented (Figure 6 and Figure 7).

### 3.3. Limitations

The major limitation in these case series arises from its small sample size and single center design. Sample size and sampling biases are inherent to individual case reports, which are not a priori-designed research studies but rather reports on the experience of individual surgeons with a novel technique. Nevertheless, case studies contribute to the early literature on a subject matter by enriching the evidence base of novel techniques and providing evidence to substantiate more robust follow-up case control studies. Moreover, longer duration follow-up will be useful in assessing outcomes, including both subjective, such as health-related-quality-of- life scores, as well as radiographic, such as instrument failure, pseudarthrosis, cage subsidence, fusion rate and adjacent segment degeneration.

## 4. Conclusions

This study highlights the importance of pre-operative assessment of sagittal balance and lumbopelvic parameters when patients present with single-level degenerative spondylolisthesis. Restoration of sagittal plane balance and lumbopelvic parameters combined with short LOS and improvement in patient reported outcomes suggest that ACR with 30° HLC can be considered when single-level spondylolisthesis is associated with high SVA at presentation. Further studies that include more patients are needed to confirm that these results are reproducible.

## Figures and Tables

**Table 1:** ACR cases demographic, clinical, and perioperative data.

Case No.	Age	Sex	Smoker	BMI	Prior ins.	EBL (mL)	ORT (min)	LOS (days)
1	67	Female	Yes	45	Yes	300	195	4
2	64	Female	No	49	No	200	190	2
3	58	Male	No	27	No	300	170	3

Abbreviations: BMI = body mass index; EBL = estimated blood loss; LOS = length of stay; ORT = operative time; Prior ins. = prior lumbar instrumentation.

**Table 2:** ACR cases sagittal balance parameters.

Case	LL° Mean	LL° SD	SVA Mean	SVA SD	PI-LL° Mean	PI-LL° SD
Pre.	−32.6	16.0	180.5	31.9	26.4	22.2
Post.	−56.0	12.8	61.3	18.0	5.1	16.5

Abbreviations: LL. = lumbar lordosis, SVA. = sagittal vertical axis measured in millimeters, PI-LL. = pelvic incidence-lumbar lordosis mismatch, Pre. = pre-operative, Post. = post-operative.

**Table 3:** ACR cases surgical data.

Case No.	Level	Arthro.	Ant ins.	Post ins.	Cage Type	Cage Size (mm)	Allograft	Autograft
1	L3-4	Yes	Yes	Yes	Expandable	11–20 × 20 × 45	Yes	Yes
2	L4-5	Yes	Yes	Yes	Expandable	11–20 × 20 × 50	Yes	No
3	L4-5	Yes	Yes	Yes	Expandable	11–20 × 20 × 55	Yes	No

Abbreviations: Ant ins. = integral anterior instrumentation, Arthro. = interbody arthrodesis, Post ins. = posterior instrumentation.

## Data Availability

Not applicable.
